# NOBLE – Flexible concept recognition for large-scale biomedical natural language processing

**DOI:** 10.1186/s12859-015-0871-y

**Published:** 2016-01-14

**Authors:** Eugene Tseytlin, Kevin Mitchell, Elizabeth Legowski, Julia Corrigan, Girish Chavan, Rebecca S. Jacobson

**Affiliations:** Department of Biomedical Informatics, University of Pittsburgh School of Medicine, The Offices at Baum, 5607 Baum Boulevard, BAUM 423, Rm 523, Pittsburgh, PA 15206-3701 USA

**Keywords:** Natural language processing, Text-processing, Named Entity Recognition, Concept recognition, Biomedical terminologies, Auto-coding, System evaluation

## Abstract

**Background:**

Natural language processing (NLP) applications are increasingly important in biomedical data analysis, knowledge engineering, and decision support. Concept recognition is an important component task for NLP pipelines, and can be either general-purpose or domain-specific. We describe a novel, flexible, and general-purpose concept recognition component for NLP pipelines, and compare its speed and accuracy against five commonly used alternatives on both a biological and clinical corpus.

NOBLE Coder implements a general algorithm for matching terms to concepts from an arbitrary vocabulary set. The system’s *matching options* can be configured individually or in combination to yield specific system behavior for a variety of NLP tasks. The software is open source, freely available, and easily integrated into UIMA or GATE. We benchmarked speed and accuracy of the system against the CRAFT and ShARe corpora as reference standards and compared it to MMTx, MGrep, Concept Mapper, cTAKES Dictionary Lookup Annotator, and cTAKES Fast Dictionary Lookup Annotator.

**Results:**

We describe key advantages of the NOBLE Coder system and associated tools, including its greedy algorithm, configurable matching strategies, and multiple terminology input formats. These features provide unique functionality when compared with existing alternatives, including state-of-the-art systems. On two benchmarking tasks, NOBLE’s performance exceeded commonly used alternatives, performing almost as well as the most advanced systems. Error analysis revealed differences in error profiles among systems.

**Conclusion:**

NOBLE Coder is comparable to other widely used concept recognition systems in terms of accuracy and speed. Advantages of NOBLE Coder include its interactive terminology builder tool, ease of configuration, and adaptability to various domains and tasks. NOBLE provides a term-to-concept matching system suitable for general concept recognition in biomedical NLP pipelines.

## Background

Natural Language Processing (NLP) methods are increasingly used to accomplish information retrieval and information extraction in biomedical systems [1]. A critical component of NLP pipelines is the matching of terms in the text to concepts or entities in the controlled vocabulary or ontology. This task is best described as ‘Concept Recognition’ although the labels ‘Entity Mention Extraction’ and ‘Named Entity Recognition’ are sometimes also used, especially among clinical NLP researchers. Ideally, such concept recognition systems produce annotations of *mentions* where the annotated term in the text may be a synonym, abbreviation, lexical variant, or partial match of the concept in the controlled vocabulary, or of the entity in the ontology. For example, given the concept “atrial fibrillation” in the terminology, we expect a concept recognition component to annotate mentions for all four of the following phrases in a text passage: ‘auricular fibrillation’ (synonym), ‘a-fib’ (abbreviation), ‘atrial fibrillations’ (lexical variant), and potentially ‘fibrillation’ (partial match). Definitions of key terms used throughout this paper are provided in Table 1.

**Table 1:** Key terms and definitions

Term	Definition
Abbreviation	A shortened form of a word, name, or phrase.
Annotation	The tagging of words comprising a mention to assign them to a concept or text feature [41].
Auto coder	A computer-based system that automatically matches text terms to a code or concept.
Concept	A “cognitive construct” that is built on our perception or understanding of something [42]; delineates a specific entity embodying a particular meaning [43].
Controlled vocabulary	A vocabulary that reduces ambiguity and establishes relationships by linking each concept to a term and its synonyms [43, 44].
Entity	An “object of interest.” [41]; the referent in the semiotic triangle.
Gazetteer	A list or dictionary of entities [45].
Lexical variant	Different forms of the same term that occur due to variations in spelling, grammar, etc. [44].
Mention	One or more words and or punctuation within a text which refer to a specific entity.
Named entity	A specific word or phrase referring to an object of interest [41, 46].
Ontology	A defined group of terms and their relationships to each other, within the context of a particular domain [47].
Semantic type	A logical category of related terms [48].
Stop word	A word of high frequency but limited information value (e.g.determiners) that is excluded from a vocabulary to improve results of a subsequent task [49].
Synonym	A term with the same meaning as another term; terms that describe the same concept [48, 50].
Term	One or more words including punctuation that represent a concept; there may be multiple terms associated with one concept [42, 49].
Terminology	A catalog of terms related to a specific domain [42]. Subsumes a variety of formalisms such as lexicons and ontologies [43].
Vocabulary	A terminology where the terms and concepts are defined [42, 44].
Word	A linguistic unit that has a definable meaning and/or function [51].

Two general approaches have been used for biomedical concept recognition [2]. Term-to-concept matching algorithms (previously called ‘auto coders’) are general-purpose algorithms for matching parsed text to a terminology. They typically require expert selection of vocabularies, semantic types, stop word lists, and other gazetteers, but they do not require training data produced through human annotation.

In contrast, machine learning NLP methods are used to produce annotators for specific well-defined purposes such as annotating drug mentions [3, 4] and gene mentions [5, 6]. Conditional random fields, for example, have produced excellent performance for specific biomedical NER tasks [4], but these systems often require training data from human annotation specific to domain and document genre. More recently, incremental approaches have been advocated for certain tasks (e.g. de-identification) [7]. These methods may be most appropriate where specific classes of mentions are being annotated [8].

For large-scale processing of biomedical documents, human annotation can be impractical because of the wide range in domains and genre represented. As a result, many current biomedical NLP pipelines and frameworks utilize some form of a general-purpose concept recognition system in combination with more specific custom annotators developed using training corpora. Commonly used general purpose concept recognition systems for biomedical tasks include MetaMap [9–11] and its Java implementation MMTx [12], MGrep [13], IndexFinder [14, 15], MedLEE [16], DOUBLET [17, 18], Concept Mapper [19], and ‘NER components’ of cTAKES [20], including the cTAKES Lucene-based Dictionary Lookup Annotator (DLA) [21] and the cTAKES Fast Dictionary Lookup Annotator (FDLA) [22]. These systems are compared for approach, availability, interoperability, and terminology source and format in Table 2. A number of these systems have been compared in other publications [23–26].

**Table 2:** Widely used concept recognition systems

System	Approach	Availability	Interoperability	Terminologies	Terminology building tools
MetaMap (and MMTx) [9–11]	Noun-phrase, lexical variants	Open Source [12, 52]	Java API for MMTx	UMLS	MetamorphoSys, DataFileBuilder
MGrep [13] (and OBA) [53]	Single word variations,	Closed Source Binary Utility	Command line utility (MGrep) integrated with RESTful API in OBA	Custom dictionaries (MGrep) with UMLS and Bioportal in OBA	N/A
	Radix-Tree search				
Concept Mapper [19]	Word Lookup Table	Open Source [54]	UIMA plugin	XML file	N/A
cTAKES Dictionary Lookup Annotator [21]	Noun-phrase, dictionary lookup	Open Source [21]	Java API with full integration in UIMA	UMLS (RRF), Bar Separated Value (BSV) file	Example scripts available [55]
cTAKES Fast Dictionary Lookup Annotator [22]	Rare Word index	Open Source [22]	Java API with full integration in UIMA	UMLS (RRF), Bar Separated Value (BSV) file	Example scripts available [55]
Index Finder [14, 15]	Word Lookup Table	N/A	N/A	UMLS	N/A
Doublet [17, 18]	Bigram Lookup Table	Open Source [56]	Command line utility (Perl)	Custom dictionary format	N/A
MedLEE [16] concept recognition	Noun-phrase	Commercial	XML based input/output	UMLS	N/A
NOBLE Coder	Word Lookup Table	Open Source [57]	Java API, UIMA and GATE wrappers	UMLS (RRF), OWL, OBO, BioPortal	Terminology Loader UI

Our laboratory has previously utilized several concept recognition systems in our NLP pipelines for processing clinical text for particular document genre (e.g. pathology reports [27]) or for specific NLP tasks (e.g. ontology enrichment [28, 29] and (in collaboration) coreference resolution [30]). In our experience, currently available systems are limited in their ability to scale to document sets in the tens and hundreds of millions, and to adapt to new document genre, arbitrary terminologies specific to particular genre, and new NLP tasks. We developed NOBLE to perform general-purpose biomedical concept recognition against an arbitrary vocabulary set with sufficient flexibility in matching strategy to support several types of NLP tasks. The system can be used for concept annotation of terms consisting of single words, multiple words, and abbreviations against a controlled vocabulary.

In this manuscript, we present the algorithmic and implementation details of NOBLE Coder and describe its unique functionality when compared with these other systems. We benchmarked the accuracy, speed, and error profile of NOBLE Coder against MMTx, MGrep, Concept Mapper, cTAKES Dictionary Lookup Annotator (DLA), and cTAKES Fast Dictionary Lookup Annotator (FDLA), all of which are general-purpose term-to-concept matching systems that have been widely used for biomedical concept recognition.

## Methods

### Algorithm

**Fig. 1:**
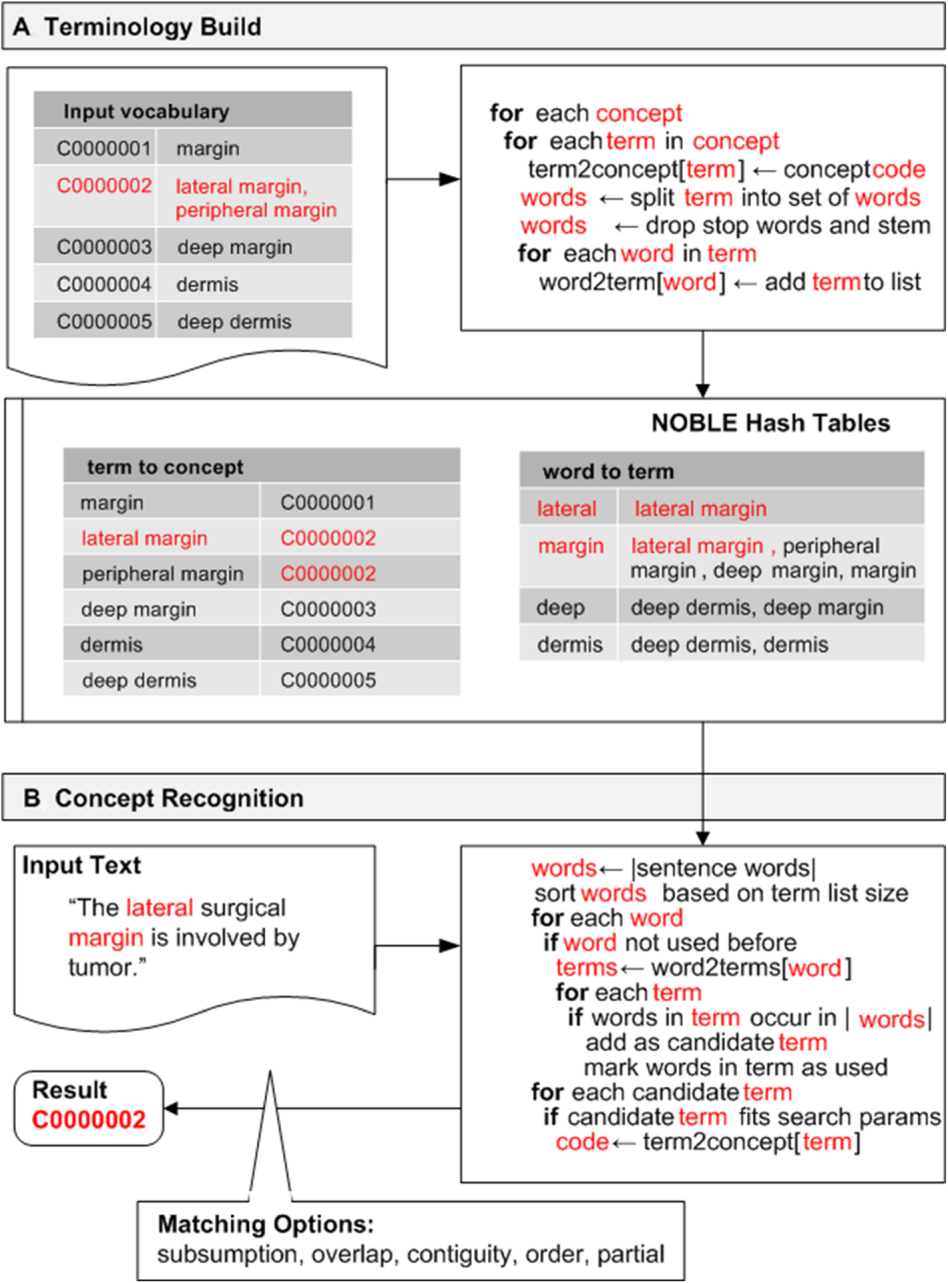
NOBLE Coder Algorithmshowing a. terminology build process and b. concept recognition process

NOBLE is a term-to-concept matching algorithm implemented using two hash tables: a word-to-terms (WT) table and a term-to-concepts (TC) table (Fig. 1). In the terminology building process (Fig. 1a), NOBLE takes a controlled vocabulary of synonymous terms, and splits each synonymous term for a given concept into component words. When the term is broken into words, each word is stemmed (Porter stemmer) to account for different inflections. This option can be disabled, which may provide slightly higher precision at the expense of recall. Words are also normalized, excluding stop words. Word normalization is performed using an approach that is similar to the method used by SPECIALIST NLP tools [31]. Each normalized term is then mapped to its corresponding concept in the TC table. In parallel, each word from a given term is mapped to a set of normalized terms that contain it in the WT table.

To perform the subsequent match (Fig. 1b), input text is broken into a set of normalized words and stop words are excluded. The word set is then ranked by frequency of associated terms. Each word is looked up in the WT table to find terms that are associated with the word and include all of the other words in the input text. This term is then added to a candidate list. Once all of the words in the input text have been processed and a set of candidate terms has been generated, each candidate term is looked up in the TC table and its concept is added to the results queue.

NOBLE has been designed to support a variety of *matching options*, which can be used to modify the output of the system for specific purposes within an NLP pipeline. These parameters, which can be altered, include (1) subsumption, (2) overlap, (3) contiguity, (4) order, and (5) partial match. Table 3 demonstrates the different concept matches (outputs) that NOBLE Coder produces based on parameter settings for a given input text and vocabulary set.

**Table 3:** NOBLE matching options with examples

Matching feature	Explanation	Example input text	Example input vocabulary	Example Output based on ParameterSetting (T, F)	
				TRUE	FALSE
Subsumption	Only more comprehensive concepts are coded	“Deep margin”	Deep	Deep margin	Deep
			Margin		Margin
			Deep margin		Deep margin
Overlap	A mapped concept may be fully or partially within the boundaries of another concept	“Deep lateral margin”	Deep	Deep	Deep
			Lateral margin	Lateral margin	Lateral margin
			Deep margin	Deep margin	
Contiguity	Words in text that map to concept term must be continuous (within word gap)	“Deep lateral margin”	Deep	Deep	Deep
			Margin	Margin	Margin
			Deep margin		Deep margin
Order	Order of words in text must be the same as in the concept term	“Margin, deep”	Deep	Deep	Deep
			Margin	Margin	Margin
			Deep margin		Deep margin
Partial	Input text that only partially matches the concept term is coded	“Margin”	Deep margin	Deep margin	No concepts coded

Matching options can be combined to create matching strategies suitable for particular NLP tasks (Table 4). If multiple candidates are generated for the same input text chunk, an optional heuristic-based scoring algorithm is applied to the results queue to select the highest-ranking candidate. Heuristics provide a method to optionally eliminate suboptimal matches that derive from specific characteristics of the terminology structure. For example, when two similar candidates are returned, NOBLE (1) prefers candidates that map to the larger number of source vocabularies, (2) rejects candidates that resemble abbreviations, but lack a case-sensitive match, and (3) prefers candidates that are un-stemmed. Using these heuristics, NOBLE respectively compensates for (1) similar concepts that have not yet been merged, (2) concepts that have abbreviation synonyms identical to common words, and (3) concepts that are incorrectly matched due to word stemming. Although this does not provide true word sense disambiguation, candidate heuristics minimize the frequency of spurious matches in our experience.

**Table 4:** Examples of NOBLE matching strategies produced by combinations of matching options

Use Cases		Combination of matching options				
Task	Description	Subsumption	Overlap	Contiguity	Order	Partial
Best match	Provides the narrowest meaningful match with the fewest candidates. Best for concept coding and information extraction.	Yes	Yes	Yes (gap = 1)	No	No
All match	Provides as many matched candidates as possible. Best for information retrieval and text mining.	No	Yes	No	No	No
Precise match	Attempts to minimize the number of false positives by filtering out candidates that do not appear in exactly the same form as in controlled terminology. Similar to best match, but increases precision at the expense of recall.	Yes	No	Yes (gap = 0)	Yes	No
Sloppy match	Allows matching of concepts even if the entire term representing it is not mentioned in input text. Best for concept coding with small, controlled terminologies and poorly developed synonymy.	No	Yes	No	No	Yes

For contiguity, the gap indicates the number of words (not counting stop words) that can occur in-between words that make up a valid term

NOBLE Coder is most reminiscent of the IndexFinder [14, 15] algorithm and system (Table 2). However, NOBLE Coder also differs from IndexFinder in several important respects, with significant consequences for its performance, scalability, and extensibility.

First, NOBLE’s terminology representation is fundamentally different from IndexFinder’s terminology representation. IndexFinder uses in-memory lookup tables with integer identifiers to look up words and terms. Constituent words are represented in a separate lookup table and string representation terms are not persisted. While this representation minimizes memory requirements, it loses information about composition of individual terms. Consequently, all words associated with a term must be counted before selecting it as a candidate. In contrast, NOBLE uses a WT table that includes the constituent words and terms (Fig. 1). This supports two major algorithmic improvements: (1) it is possible to avoid looking up a list of terms associated with words that are already present in previously selected candidate terms; and (2) the algorithm can first consider more informative words with fewer associated terms, because the number of terms associated with each word in the WT table is known.

To increase processing speed, NOBLE Coder creates a cache of the word-to-term table that contains 0.2 % of the most frequently occurring words in a dictionary. This table contains a reduced set of terms that include only the cached words. NOBLE determines if a word is cached before using the main WT table. The threshold was empirically chosen based on a series of experiments comparing a range of thresholds.

Second, NOBLE Coder uses hash tables to represent the WT table, and relies on the underlying JVM to handle references to string objects internally. NOBLE Coder also uses JDBM (Java Database Management) [32] technology to optionally persist those hash tables to disk. This enables NOBLE to input a terminology of any size, since it does not need to fit into memory. IndexFinder leverages a sorted table of run-length identifiers, and therefore trades complexity at build time for a smaller memory footprint. In contrast, NOBLE Coder stores a list of term sizes, requiring more memory but eliminating the requirement to load the entire vocabulary each time the system is initialized.

Third, NOBLE Coder is arguably more extensible and customizable when compared with IndexFinder. NOBLE supports multiple matching strategies enabling the optional inclusion or exclusion of candidates based on (1) subsumption, (2) overlap, (3) contiguity, (4) order, and (5) partial match. NOBLE has been implemented to support a variety of terminology import formats including RRF, OWL, OBO, and Bioportal. The published implementation of IndexFinder was specific to UMLS.

Finally, NOBLE supports dynamic addition and subtraction of concepts to the terminology while processing. This feature can be used as part of a cycle of terminology enrichment by human reviewers or by automated methods.

## Implementation

NOBLE Coder is implemented in Java and is part of a suite of Java NLP tools developed at University of Pittsburgh. In addition to the WT and TC tables described earlier, NOBLE Coder uses additional tables to store word statistics, terminology sources, and concept metadata such as hierarchy information, definitions, semantic types, and source vocabularies. NOBLE Coder uses JDBM 3.0 library as a NoSQL solution to persist these tables to disk.

NOBLE Coder supports several additional features and tools that enhance its utility and usability. These features include (1) matching regular expressions, (2) resolving acronyms to their expanded terms, (3) filtering concepts by source or semantic types, (4) skipping problem words, and (5) selecting a single candidate for a matched term. Another feature that expands usability is the User Interface (UI) for running the concept recognition system and generating results as both tab-delimited files and interactive annotated documents in HTML format.

**Fig. 2:**
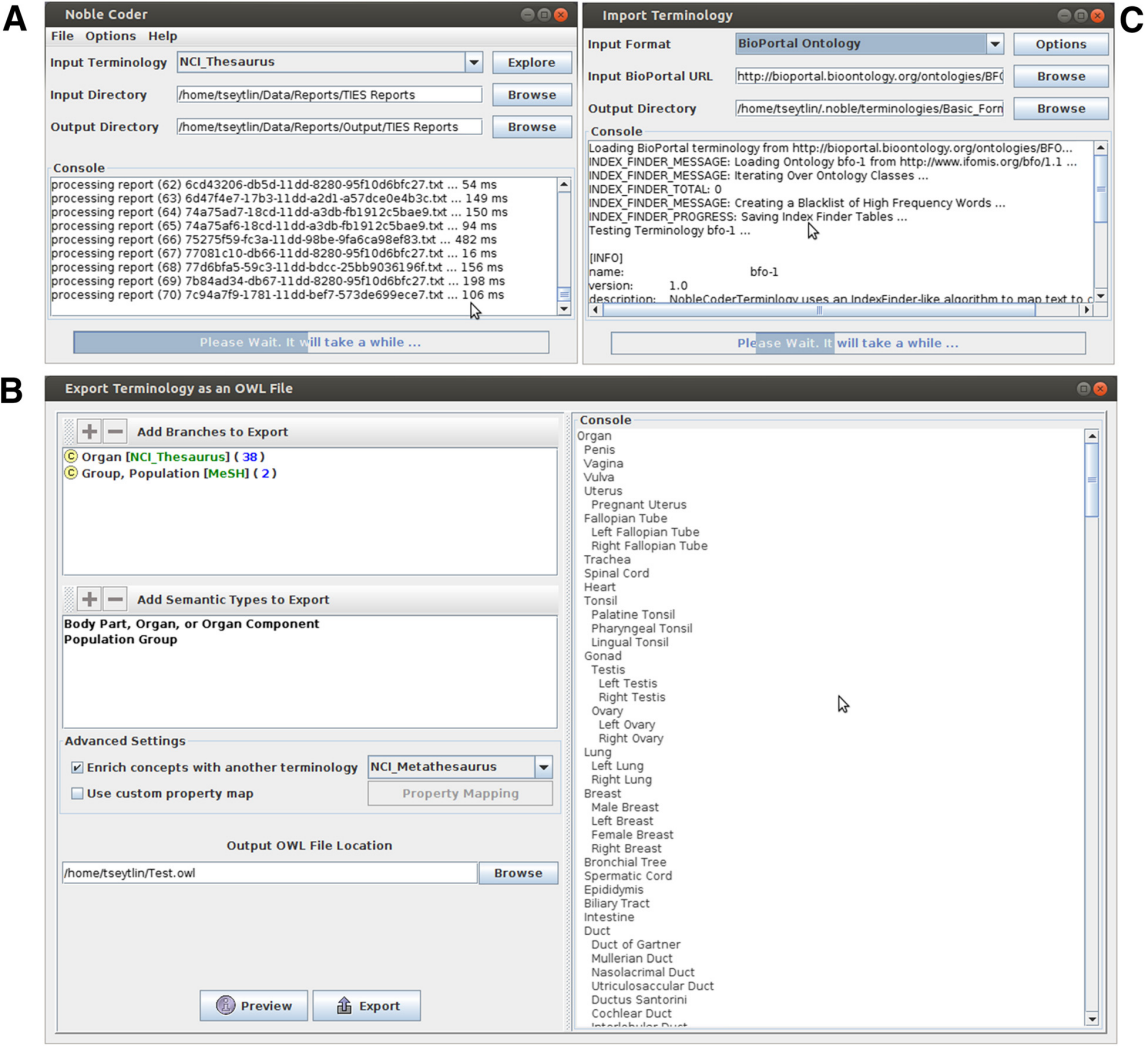
NOBLE Coder User Interface. **a** Example of NOBLE Coder processing reports. **b** Terminology Importer loads the Bioporter ontology, one of the many supported formats. **c** Terminology Exporter creates a custom terminology by merging branches from two different terminologies

NOBLE Coder has also been designed to minimize the amount of user effort required to input terminologies, including ontologies. For example, NOBLE Coder uses a bundled Terminology Importer UI so users can easily import custom terminologies in multiple formats (RRF, OWL, OBO, and BioPortal). NOBLE Coder also allows users to select multiple branches from a set of terminologies, filter by semantic types, and export the results as an OWL file. Furthermore, users can visually and programmatically browse taxonomic relationships in a loaded terminology (Fig. 2).

NOBLE Coder is open source and freely available for academic and non-profit use under the terms of the GNU Lesser General Public License v3. Source code, documentation, and executable, with UIMA and GATE wrappers, are available at https://sourceforge.net/projects/nobletools/. For rapid evaluation, users may run NOBLE Coder in Java Webstart at http://noble-tools.dbmi.pitt.edu/noblecoder.

We have used NOBLE Coder as part of a new NLP pipeline for TIES v5.0 [33] used to processed more than 25 million de-identified clinical documents. The TIES system [27] is used by clinical and translational researchers at our institution and other institutions across the country.

### Benchmarking

We compared NOBLE Coder to five widely used, general-purpose concept recognition systems: MGrep, MMTx, Concept Mapper, cTAKES Dictionary Lookup Annotator (DLA), and cTAKES Fast Dictionary Lookup Annotator (FDLA). Because NOBLE Coder was originally written to replace MMTx in our TIES system, we chose to evaluate MMTx rather than its Prolog analog, MetaMap. As described by its NLM developers [9], MMTx is a Java implementation of MetaMap, which produces only minor differences in comparison. These discrepancies result in large part from MMTx’s tokenization and lexicalization routines. MMTx continues to be widely used throughout the biomedical informatics community, in part because of (1) its simpler installation using a single server and (2) its more flexible vocabulary building process.

**Fig. 3:**
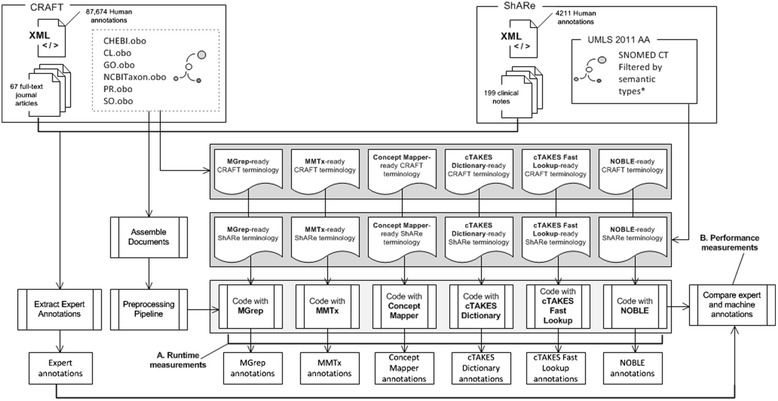
Benchmarking Study

We used two human annotated corpora as a basis for performance comparisons. Evaluation included measurements of run time speed (Fig. 3a) and standard performance metrics (Fig. 3b), as well as an analysis of false positive and false negative errors made by NOBLE Coder on both corpora. In order to minimize the differences among implementations, we performed all tests in a standard NLP framework, replacing only the NER processing resources between runs.

#### System versions and configurations

Systems included in this benchmarking study differed considerably in the number and types of parameters, as well as the extent to which those parameters could be manipulated. In selecting parameter settings for each system, we sought to achieve uniform behavior that best captured the annotation guidelines associated with the reference standards [34, 35]. In addition, we favored settings that, in our experience, have shown superior concept recognition for an overall information extraction strategy. Previous studies of concept recognition systems using the Colorado Richly Annotated Full Text (CRAFT) corpus have shown that adjusting parameters can produce significant variation in performance across corpora, terminology, and terminology subset [26]. Rather than taking an exhaustive approach, our goal was to replicate the “best match” strategy, which is a common use case for concept recognition in information extraction tasks. Consequently, we set parameters to achieve *Overlap*, *Contiguity*, and *Subsumption* (Table 3), which are equivalent to the NOBLE Coder ‘best match’ strategy. Selected parameters for each concept recognition system are shown in Table 5. We relate each given parameter to the associated general behavior achieved, which are mirrored by named NOBLE Coder matching options. Standard settings that were not adjusted are not shown.

**Table 5:** Parameter settings used for concept recognition systems

Concept recognition system	Download reference	Parameters changed from standard	Goal achieved
MMTx	[12]	Best-mapping = false	Overlap
MGrep	[58]	Longest match = true	Subsumption
Concept Mapper	[59]	Contiguous match = true	Contiguity
cTAKES Dictionary Lookup Annotator	[21]	N/A	N/A
cTAKES Fast Dictionary Lookup Annotator	[22]	OverlapJCasTermAnnotator	Overlap
		PrecisionTermConsumer	Subsumption
NOBLE Coder	[57]	Best Match Strategy	Overlap
			Contiguity
			Subsumption

To minimize differences due to platform, all systems were run in the context of a cTAKES UIMA pipeline that included standard front-end cTAKES Text Annotators for Tokenization and Sentence Detection. Each coder was entirely wrapped as a UIMA Annotator, with the exception of MGrep. MGrep was partially wrapped as a UIMA Annotator as described below.

#### Corpora and vocabularies

We used two publicly available, human-annotated corpora for comparison of the five systems. These corpora were selected because they represent very different tasks (clinical text annotation versus biomedical literature annotation) and very different vocabularies (OBO ontologies versus SNOMED-CT), allowing us to better assess generalizability during the benchmarking process. For each corpus vocabulary, we built annotator-ready vocabulary footprints as described below.

##### The Colorado Richly Annotated Full Text (CRAFT) corpus

The Colorado Richly Annotated Full Text (CRAFT) Corpus [35] is a publicly available, human annotated corpus of full-text journal articles from a variety of disciplines. Articles focus on mouse genomics. All articles are from the PubMed Central Open Access Subset. Human concept annotations are provided as Knowtator XML files with standoff markup. The evaluation set contained a total of 67 document, 438,705 words, and 87,674 human annotations.

Machine annotations by all five systems were generated using the terminologies and version provided with the CRAFT corpus (CHEBI.obo, CL.obo, GO.obo, NCBITaxon.obo, PR.obo and SO.obo). Based on published recommendations by the corpus developers [36], we elected to exclude Entrez gene from the human annotations. Therefore, we did not utilize the Entrez gene vocabulary, relying instead on the PR ontology for gene mentions.

##### The Shared Annotated Resources (ShARe) corpus

We obtained the Shared Annotated Resources (ShARe) corpus [34] under a Data Use Agreement with the Massachusetts Institute of Technology (MIT). The ShARe corpus consists of de-identified clinical free-text notes from the MIMIC II database, version 2.5, that was used for the SemEval 2015 challenge [37]. Document genres include discharge summaries, ECG (electrocardiogram) reports, echocardiogram reports, and radiology reports. Human annotations are provided as Knowtator XML files with standoff markup. For the purposes of this study, we used the entire training set of documents. The evaluation set contained a total of 199 documents, 94,243 words, and 4,211 human annotations.

Machine annotations by all five systems were generated using the same terminology, version, and semantic types as designated by the ShARe schema [37, 38]. SNOMED CT was obtained from UMLS version 2011AA and filtered by semantic type to include only Congenital Abnormality, Acquired Abnormality, Injury or Poisoning, Pathologic Function, Disease or Syndrome, Mental or Behavioral Dysfunction, Cell or Molecular Dysfunction, Experimental Model of Disease, Anatomical Abnormality, Neoplastic Process, and Signs and Symptoms.

#### Vocabulary build process

For all concept recognition systems except NOBLE Coder, significant additional effort was required to import the vocabulary into a format that could be used by the system. Additionally, some systems only worked when the concept code was in the form of a UMLS CUI string (e.g. cTAKES DLA and FDLA), while others (e.g. MGrep) expected integers. In order to standardize formats and to account for these different representations, we used UMLS Rich Release Format (RRF) as the least common denominator for all vocabulary builds. We note that NOBLE already ingests many different formats (including OBO, OWL, BioPortal and RRF). For the sake of consistency, we utilized RRF as the input format for NOBLE Coder too.

To construct coder-ready terminologies from CRAFT, we developed Java code to convert OBO to Rich Release Format (RRF). These transformational tools are also made publicly available at https://sourceforge.net/projects/nobletools/. We generated UMLS-like identifiers in a cui-to-code mapping table to enable subsequent comparison to the gold annotations.

To construct coder-ready terminologies for ShARe, we used UNIX command line tools (grep and awk) to extract SNOMED CT terminology, filter to the required semantic types [34], and output RRF.

NOBLE Coder ingests native RRF files as one of its input formats. No other transformations were performed. Subsequent manipulations required for all other systems are described in Table 6.

**Table 6:** Vocabulary build steps required for each system

Concept Recognition Systems	Dictionary Data Structure Used by Coder
MMTx^a^	Used MetamorphoSys to convert RRF to ORF and used bundled data file builder to create terminology for each corpus; this process required significant user interaction and took many hours
MGrep	Sent RRF files for both corpora to the MGrep authors and received from them a tab delimited text file that could be used with the MGrep system enriched with LVG; there is limited publicly available information about the vocabulary format required by MGrep
Concept Mapper	Wrote custom Java code to convert RRF files to an XML file formatted in the Concept Mapper valid syntax
cTAKES Dictionary Lookup Annotator	Wrote custom Java code to convert RRF files to seed a Lucene Index
cTAKES Fast Dictionary Lookup Annotator	Wrote custom Java code to convert RRF into Bar Separated Values (BSV) file that FDLA imports
NOBLE Coder^a^	Directly imported RRF files

^a^Systems that have vocabulary import and selection tooling

#### Document processing

Expert annotations for both corpora were published as Knowtator XML files. We used the Apache XMLDigester package to read these files and transform their content to a set of comma separated value (CSV) files containing the annotation span, and concept unique identifier. Natural Language Processing was performed with the cTAKES UIMA Processing Pipeline (Fig. 3). Documents were initially pre-processed using cTAKES off-the-shelf Tokenizer and Sentence Splitter modules. They were then processed with each of the individual concept recognition components. Each concept recognition system generated a set of concept annotations that were converted into a CSV format matching the format of the gold annotation set.

##### Measurement of run times

Run time was defined as the time to process all documents in the corpus (preprocessing and coding), as reported by the NLP Framework Tools. Each concept recognition system was run ten times in the context of a simple UIMA pipeline. Measurements were made on a single PC with Intel I7 × 8 processor and 32 GB of RAM, running Ubuntu 12.04. No significant competing processes were running. A total of 120 runs were performed representing each of the six coders, across both corpora, over ten runs. Runs were randomly sequenced to minimize order effect. Resources were freed and garbage was collected before each new run was started. Results are reported as median milliseconds with interquartile range (IQR).

Because MGrep runs as a command line program, we used a two-step process for MGrep in which we first produced a set of annotations cached in JDBM (now MapDB) [32], and then wrapped a series of hash map lookups with a UIMA Annotator. Run times for MGrep were calculated as the sum of these two steps. In this way, we avoided overly penalizing MGrep for start-up time, which would be incurred with each text chunk.

##### NLP performance metrics

Standard NLP evaluation metrics were used, including TP, FP, FN, Precision, Recall, and F-1 measure. The quality of the software annotations was determined via comparison to the Expert Annotation Set using GATE’s AnnotDiffTool [39], which computes a standard Precision, Recall, and F-1 measure using True Positive, False Positive, and False Negative distributions. We ran in *average mode*, which also considers partial positives (PP). PPs are defined as any inexact overlap between the software proposed annotation and the expert annotation. Scoring with *average mode* allocates one-half the Partial Positive count into both the Precision and Recall calculations.

##### Error analysis

To evaluate the overall error profile of NOBLE Coder against other systems, we examined the distribution of all errors (both false positive and false negative) made by NOBLE Coder and determined when these specific NOBLE Coder errors were also made by one or more of the other systems. From this set of all NOBLE Coder errors, we then randomly sampled 200 errors made by NOBLE Coder on each corpus, including 100 false positives and 100 false negatives and yielding a total of 400 errors. Each error in this sampled set was then manually classified by a human judge to determine the distribution of errors based on 10 categories of error types. Categories included errors that could result in false positives (e.g. word sense ambiguity), false negatives (e.g. incorrect stemming), or both false positives and false negatives (e.g. boundary detection). Our categories built on previous error categories used in a study of MMTx [40], which we further expanded. All 400 errors studied were classified as one of these error types. We then analyzed the frequency with which these specific NOBLE Coder errors were also made by the other systems. For error analyses, we considered only absolute matches and not partial positives, which would have significantly complicated the determination of overlap in errors among all six systems.

## Results

All systems performed better on the ShARe corpus than they did on the CRAFT corpus (Table 7). There are likely various factors that contribute to the observed differences. The CRAFT corpus contains a large number of gene and protein mentions, often with complex combinations of letters, words, numbers, and punctuation. Such a terminology is more prone to ambiguity between entries or with common English terms. Behavior was most disparate for MGrep, which was four times more accurate on the ShARe corpus. Because MGrep does not consider all word orders, it can be expected to perform better with vocabularies that include different orderings of multi-word terms as synonyms. MGrep’s vocabulary build process also includes UMLS lexical variant generation, which could be expected to improve performance on the ShARe corpus more than on the CRAFT corpus.

**Table 7:** Performance metrics

Corpus	Concept Recognition System	TP	PP	FP	FN	Precision	Recall	F1	Median runtime over 10 runs (ms)**	IQR (ms)
CRAFT	MMTx	35,140	659	45,791	51,875	0.43	0.40	0.42	640,450	3,937
CRAFT	MGrep	9,955	292	10,666	77,427	0.48	0.12	0.19	27,448*	747
CRAFT	Concept Mapper	29,353	713	32,122	57,608	0.48	0.34	0.40	5,329	113
CRAFT	cTAKES Dictionary Lookup	37,736	742	36,951	49,196	0.51	0.43	0.47	4,082,685	3,459
CRAFT	cTAKES Fast Lookup	35,078	784	51,383	51,812	0.41	0.4	0.41	9,812	1,160
CRAFT	NOBLE Coder	36,568	1,637	46,344	49,469	0.44	0.43	0.43	17,431	44
ShARe	MMTx	2,375	101	1,675	1,735	0.58	0.58	0.58	52,016	2,678
ShARe	MGrep	2,340	35	1,075	1,836	0.68	0.56	0.62	7,103*	148
ShARe	Concept Mapper	2,302	34	2,483	1,875	0.48	0.55	0.51	1,543	57
ShARe	cTAKES Dictionary Lookup	2,417	39	2,587	1,755	0.48	0.58	0.53	263,336	2,316
ShARe	cTAKES Fast Lookup	2,374	36	1,101	1,801	0.68	0.57	0.62	2,754	81
ShARe	NOBLE Coder	2,315	99	1,413	1,797	0.62	0.56	0.59	6,466	94

^*^MGrep runtime is a sum of the runtime of a harness and a stand-alone MGrep invocation on a corpus

**all measurements were performed on a UIMA platform and a Linux Workstation, 32GB RAM, Intel® Core™ i7-3770 CPU @ 3.40GHz

On CRAFT, NOBLE Coder achieved an F^1^ value of 0.43, ranking second behind the cTAKES Dictionary Lookup Annotator (F^1^ = 0.47), and slightly better than MMTx (F^1^ = 0.42), cTAKES Fast Dictionary Lookup Annotator (F^1^ = 0.41), and Concept Mapper (F^1^ = 0.40). All five of these coders markedly outperformed MGrep (F^1^ = 0.19).

On ShARe, NOBLE Coder achieved an F^1^ value of 0.59, ranking third behind cTAKES Fast Dictionary Lookup Annotator (F^1^ = 0.62) and MGrep (F^1^ = 0.62) which were tied. NOBLE Coder was slightly more accurate than MMTx (F^1^ = 0.58), and performed better than cTAKES Dictionary Lookup Annotator (F^1^ = 0.53), and Concept Mapper (F^1^ = 0.51).

With respect to speed, Concept Mapper was the fastest on both corpora, followed by cTAKES Fast Dictionary Lookup Annotator and NOBLE Coder. All three of the fastest annotators completed the CRAFT corpus with a median run speed of less than twenty seconds, and the ShARe corpus with a median run speed of less than seven seconds. MGrep was only slightly slower than NOBLE Coder was and completed annotations of the CRAFT and ShARe corpora in 27.5 and 7.1 s, respectively. In contrast, MMTx completed the annotation of the CRAFT corpus in 10 min and the ShARe corpus in 1 min. cTAKES Dictionary Lookup Annotator was the slowest on both corpora, completing the annotation of the CRAFT corpus in 68 min and the ShARe corpus in 4.3 min.

**Fig. 4:**
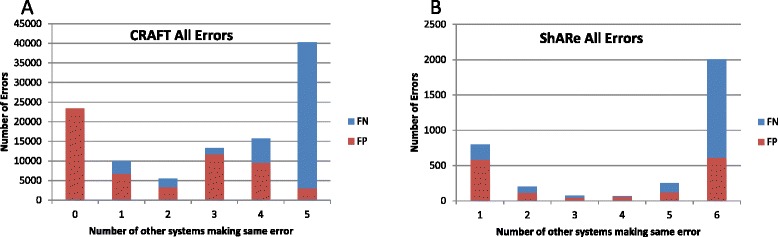
Frequency of All Errors Made by NOBLE Coder and Other Systems Shows the total number of errors made by NOBLE Coder and the number of other systems that made the same errors. **a** Total number of FN and FP errors on CRAFT corpus. **b** Total number of FN and FP errors on ShARe corpus

We investigated the frequency of errors made by NOBLE Coder on both corpora and then determined the distribution of these errors across the other systems. As shown in Fig. 4, for both corpora, there is substantial overlap among FN errors, with the same missed entities being missed by multiple systems. Of note, most of the errors made by NOBLE Coder were made by at least three other systems. As would be expected, FP errors have less overlap. However, once again, errors made by NOBLE Coder are frequently made by the other coders, with the majority of NOBLE Coder FP errors made by three or more systems.

The distribution of errors made by NOBLE Coder on a set of randomly sampled FN and FP errors is shown in Table 8. For both corpora, the most frequent type of error related to absence of background knowledge or context when compared to the human annotated gold corpus. For example, in the ShARe corpus, the phrase “No W” appears in the review of systems within the documentation of the physical examination. “W” was annotated by the humans as a mention of the concept “wheezing” (C0043144). The human annotators understood the context of the highly condensed list of clinical pertinent negatives used, and they also knew the discourse-specific abbreviations that were used in the review of systems for pertinent negatives in auscultation. “W” is not a synonym for “wheezing” in the vocabulary, and NOBLE lacks the contextual understanding of such abbreviations. NOBLE Coder therefore missed this mention. NOBLE Coder also annotated entities that were not annotated by the expert human annotators, even though the mentions annotated appear to be reasonable concept annotations. For example, in the CRAFT corpus the phrase “Cells were lysed in Laemli buffer” appears. NOBLE Coder annotated “buffer” with the concept “buffer” (CHEBI:35225), but no matching human annotation appears in the gold corpus. Such entities may not have been deemed sufficiently important to merit annotation, based on the annotation guidelines.

**Table 8:** Analysis of sampled NOBLE Coder errors

Error Type	Definition	Type of error	CRAFT	ShARe
Boundary detection	Incorrectly incorporates words from earlier or later in the sentence, considering them to be part of the concept annotated	FP	0 (0 %)	3 (1.5 %)
Concept hierarchy	Incorrectly assigns more general or more specific concept than gold standard	FP and FN	18 (9 %)	13 (6.5 %)
Context/background knowledge	Concept annotated incorrectly because context or background knowledge was needed	FP and FN, usually FN	72 (36 %)	81 (40.5 %)
Exact match missed	Concept not annotated despite exactly matching the preferred name or a synonym	FN	2 (1 %)	3 (1.5 %)
Importance	Annotated concept was not deemed relevant by gold annotators	FP	33 (16.5 %)	51 (25.5 %)
Abbreviation detection	Abbreviation defined in the dictionary had a case-insensitive match, because it did not match a defined abbreviation pattern	FP	18 (9 %)	0 (0 %)
Alternative application of terminology	Gold used obsolete term, term is not in SNOMED, or same term existed in multiple ontologies, resulting in different annotations for same mention	FN	31 (15.5 %)	10 (5 %)
Text span	Concept annotated was identical to gold but text span was different than gold	FP and FN	10 (5 %)	20 (10 %)
Word sense ambiguity	Concept annotated was used in different word sense	FP	4 (2 %)	0 (0 %)
Wording mismatch	Missing or incorrect annotation due to word inflection mismatch between dictionary term and input text	FP and FN, usually FN	12 (6 %)	19 (9.5 %)
Total Errors			200 (100 %)	200 (100 %)

Among the errors sampled from the CRAFT corpus, other error types identified included (with decreasing frequency) an alternative application of the terminology, concept hierarchy errors, abbreviation detection errors, wording mismatch, text span mismatch, word sense ambiguity, and missed exact matches. Among the errors sampled from the ShARe corpus, other error types identified included (with decreasing frequency) text span mismatch, wording mismatch, concept hierarchy errors, an alternative application of the terminology, and boundary detection.

**Fig. 5:**
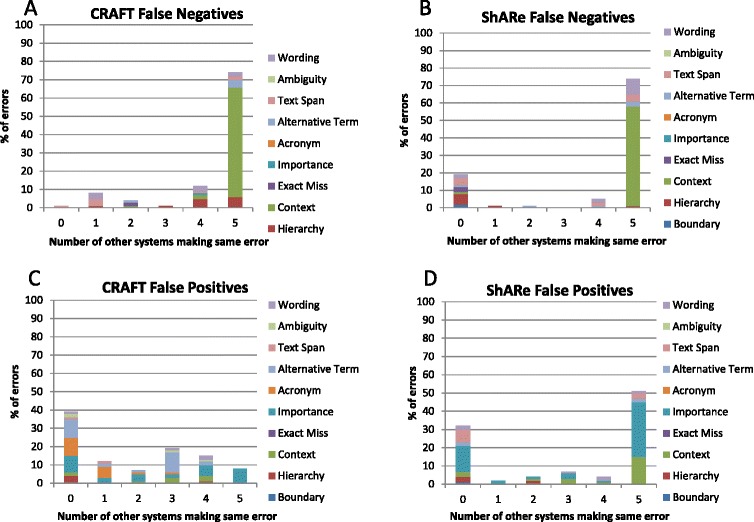
Frequency of Error Types Made by NOBLE Coder and Other Systems Shows the number of FP and FN errors made by NOBLE Coder on the CRAFT and ShARe corpora, and the number of other systems that made the same errors. **a** Number of FN errors on CRAFT corpus. **b** Number of FN errors on ShARe corpus. **c** Number of FP errors on CRAFT corpus. **d** Number of FP errors on ShARe corpus

Finally, we looked at the overlap of error types among other systems for the 400 sampled NOBLE Coder errors (Fig. 5). Not surprisingly, NOBLE Coder FN errors that were judged to be related to context were commonly seen in all other systems. Other errors were largely evenly distributed.

## Discussion

In this manuscript, we introduce NOBLE Coder, a concept recognition system for biomedical NLP pipelines. The underlying algorithm of NOBLE Coder is based on a word lookup table, making it most similar to the IndexFinder [14, 15] and Concept Mapper [19] systems. However, NOBLE Coder is different from these systems in a number of respects, with consequences for its performance, scalability, and extensibility (Table 1).

Our benchmarking tests showed that NOBLE Coder achieved comparable performance when compared to widely used concept recognition systems for both accuracy and speed. Our error analysis suggests that the majority of the NOBLE Coder errors could be related to the complexity of the corpus, expert inferences made by the annotator, annotation guideline application of the human annotators, and missing (but reasonable) annotations created by NOBLE Coder that were not identified by the gold annotators. Errors due to known weaknesses of concept recognition systems (such as word sense ambiguity and boundary detection) occurred relatively infrequently in both corpora.

Overall, NLP performance metrics (precision, recall, and F^1^) were low for all systems, particularly on the CRAFT corpus, which was not unexpected. Results of our component benchmarking tests should not be directly compared to evaluations of entire systems, including those tested against these corpora with reported results in the high 70s F-score [37, 38]. We specifically sought to separate out and test the concept recognition system alone without the use of noun phrasing, part of speech tagging, pre-processing of the vocabulary, or filtering results to specific semantic types. When concept recognition is used within the context of a pipeline that performs these other functions, performance can be expected to be higher.

Nevertheless, our results provide additional data to other comparative studies by evaluating recall, precision, and speed for all of these systems across two very different gold standards. No previous study has compared performance on more than one reference standard. Our results demonstrate that some systems generalize more easily than others do. Additionally, no previous study has directly compared the cTAKES Dictionary Lookup Annotator and Fast Dictionary Lookup Annotator with other alternatives. Our results demonstrate the remarkable speed of the cTAKES Fast Dictionary Lookup Annotator with increased accuracy on the ShARe corpus.

The results of our benchmarking tests are consistent with findings of other recent studies that demonstrate the greater precision and greater speed of MGrep over MetaMap [23–25]. However, the low recall of MGrep, for example on the CRAFT corpus, may limit its utility to tasks in which a UMLS-derived vocabulary is used with lexical variant generation. To evaluate speed fairly across systems, we ran all systems within the context of a cTAKES UIMA pipeline. The command line version of MGrep can be expected to run at faster speeds.

A recent study of concept recognition systems using the CRAFT corpus showed that Concept Mapper achieved higher F^1^ measures when compared to both MetaMap and NCBO annotator (which implements MGrep) across seven of eight ontologies represented in the gold set annotations [26]. We did not specifically compare accuracy against specific ontology subsets, and therefore our results are not directly comparable.

NOBLE Coder has some similarities to Concept Mapper including the use of a word-to-term lookup table and the ability to create different matching strategies. But there are also a large number of differences between the systems, including NOBLE Coder’s use of JDBM, native imports of vocabularies as NoSQL tables, and additional parameters for setting the mapping strategy (e.g. word order and partial match) when compared with ConceptMapper. A particularly important difference is that the NOBLE algorithm is greedy, which is likely to improve performance with very large terminology sets and corpora.

NOBLE Coder also has significant advantages over in-memory systems. Use of heap memory is an advantage if the system will be run on 32-bit machines or in any way that is RAM limited. Disk-based concept recognition systems such as NOBLE Coder, MMTx, and disk-configured deployments of cTAKES DLA and FDLA would likely run with smaller footprints than their in-memory counterparts. Additionally, NOBLE Coder can enable the application programmer to directly manage heap growth, with its utilization of MapDB just-in-time caching technology. None of the other systems has this capability. Memory management aspects of NOBLE make it an excellent choice for NLP processing in cloud-based environments where higher RAM requirements also increase cost.

In addition to performance metrics and speed, usability within common NLP tasks and scenarios is an important criterion to consider when selecting processing resources for an NLP pipeline. We observed significant differences in the degree of effort required to achieve a specific behavior such as Best Match. Systems that expose and explicitly document parameters that can be altered to achieve such behaviors may be preferred for some applications. The terminology build process remains one of the most complex and challenging aspects of working with any general concept recognition system. Of the systems described in Table 1 that support more than a single pre-determined vocabulary, only MMTx and NOBLE provide both tooling and documentation to build the necessary terminology resources. Most systems also have significant limitations in the formats and types of vocabularies that can be used. In large part, NOBLE was developed to address this limitation. It currently supports a variety of terminology import formats including RRF, OWL, OBO, and Bioportal. Finally, of the systems tested, only NOBLE supports dynamic addition and subtraction of concepts to the terminology while processing. This feature can be used as part of a cycle of terminology enrichment by human reviewers or by automated methods.

Results of our benchmarking tests may provide guidance to those seeking to use a general-purpose concept recognition system within the context of their biomedical NLP pipelines. Given specific use cases, individual researchers must prioritize not only (1) precision, (2) recall, and (3) speed, but also (4) generalizability, (5) ability to accommodate specific terminologies and (6) terminology formats, and (7) ease of integration within existing frameworks. The reader should also be aware that any general-purpose annotator must ultimately be evaluated within the context of a larger pipeline system.

## Conclusions

NOBLE Coder is an open source software component that can be used for biomedical concept recognition in NLP pipelines. We describe the system and show that it is comparable to the highest performing alternatives in this category. NOBLE Coder provides significant advantages over existing software including (1) ease of customization to user-defined vocabularies, (2) flexibility to support a variety of matching strategies and a range of NLP tasks, and (3) disk-based memory management, making the software particularly well suited to cloud hosted environments.

## Availability and requirements

Project name: NOBLE Coder and NOBLE Tools

Project home page: http://noble-tools.dbmi.pitt.edu/

Operating system(s): Linux, Windows, MacOS

Programming language: Java

Other requirements: None; can be used as standalone application, or within UIMA or GATE

License: Gnu LGPL v3

Any restrictions (to use by non-academics): University of Pittsburgh holds the copyright to the software. Use by non-profits and academic groups is not restricted. Licensing for use by a for-profit commercial entity may be negotiated through University of Pittsburgh Office of Technology Management.
